# Factors associated with SARS-CoV-2-related hospital outcomes among and between persons living with and without diagnosed HIV infection in New York State

**DOI:** 10.1371/journal.pone.0268978

**Published:** 2022-05-25

**Authors:** Elizabeth M. Rosenthal, Eli S. Rosenberg, Wendy Patterson, Wendy P. Ferguson, Charles Gonzalez, Jack DeHovitz, Tomoko Udo, Deepa T. Rajulu, Rachel Hart-Malloy, James Tesoriero

**Affiliations:** 1 New York State Department of Health, Albany, New York, United States of America; 2 University at Albany School of Public Health, State University of New York, Rensselaer, New York, United States of America; 3 Center for Collaborative HIV Research in Practice and Policy, University at Albany School of Public Health, State University of New York, Rensselaer, New York, United States of America; 4 IPRO, Lake Success, New York, United States of America; 5 Downstate Health Sciences University, State University of New York, Brooklyn, New York, United States of America; Tanta University Faculty of Medicine, EGYPT

## Abstract

**Background:**

Persons living with diagnosed HIV (PLWDH) are at increased risk for severe illness due to COVID-19. The degree to which this due to HIV infection, comorbidities, or other factors remains unclear.

**Methods:**

We conducted a retrospective matched cohort study of individuals hospitalized with COVID-19 in New York State between March and June 2020, during the first wave of the pandemic, to compare outcomes among 853 PLWDH and 1,621 persons without diagnosed HIV (controls). We reviewed medical records to compare sociodemographic and clinical characteristics at admission, comorbidities, and clinical outcomes between PLWDH and controls. HIV-related characteristics were evaluated among PLWDH.

**Results:**

PLWDH were significantly more likely to have cardiovascular (matched prevalence-ratio [mPR], 1.22 [95% CI, 1.07–1.40]), chronic liver (mPR, 6.71 [95% CI, 4.75–9.48]), chronic lung (mPR, 1.76 [95% CI, 1.40–2.21]), and renal diseases (mPR, 1.77 [95% CI, 1.50–2.09]). PLWDH were less likely to have elevated inflammatory markers upon hospitalization. Relative to controls, PLWDH were 15% less likely to require mechanical ventilation or extracorporeal membrane oxygenation (ECMO) and 15% less likely to require admission to the intensive care unit. No significant differences were found in in-hospital mortality. PLWDH on tenofovir-containing regimens were significantly less likely to require mechanical ventilation or ECMO (risk-ratio [RR], 0.73 [95% CI, 0.55–0.96]) and to die (RR, 0.74 [95% CI, 0.57–0.96]) than PLWDH on non-tenofovir-containing regimens.

**Conclusions:**

While hospitalized PLWDH and controls had similar likelihood of in-hospital death, chronic disease profiles and degree of inflammation upon hospitalization differed. This may signal different mechanisms leading to severe COVID-19.

## Introduction

As Coronavirus Disease 2019 (COVID-19) became one of the leading causes of mortality in the U.S. [1], understanding whether people living with diagnosed HIV (PLWDH) may be particularly vulnerable to severe COVID-19 illnesses became an important area of research due to inferred immunocompromised nature of HIV [2]. Using New York State (NYS) HIV and COVID-19 surveillance data, we previously reported more severe COVID-19 outcomes, including higher rates of hospitalization and mortality among PLWDH compared to non-PLWDH in NYS [3]. This study, however, did not include clinical features of COVID-19 due to limitations associated with use of surveillance data. Prior studies investigating clinical features of COVID-19 in PLWDH showed inconsistent results and with small sample sizes [4–12]. Larger studies are needed to fully explore the clinical outcomes of COVID-19 among PLWDH, and associated risk factors.

In addition to being immunocompromised, high prevalence of several chronic conditions, such as chronic kidney disease, cardiovascular disease, obesity, and type 2 diabetes mellitus [11, 13–15], are an important potential pathway to severe COVID-19 illnesses in PLWDH [13, 16, 17]. Studies often focus on an independent association between HIV infection and COVID-19 outcomes by adjusting for comorbidities, including a recent study by the World Health Organization (WHO) that reported significantly greater likelihood of in-hospital mortality and severe COVID-19 in PLWDH relative to non-PLWDH patients [18]. However, such adjustments may leave residual confounding due to factors like healthcare system capacity and structure being largely unaccounted for and, thus, fail to accurately assess the true risk for severe COVID-19 illnesses among PLWDH. Controls that are matched on key demographic characteristics, place and time of hospitalization help address these confounding factors.

Furthermore, patients with low CD4 cell count or unsuppressed HIV may be particularly vulnerable to severe COVID-19 outcomes [19–23], though our previous study [3] found significantly elevated risk of hospitalization relative to non-PLWDH even among PLWDH who were virally suppressed and had a CD4 count above 500 cells/mm^3^ at their last HIV-related laboratory exam (i.e., presumably the healthiest PLWDH). We also found a higher hospitalization risk with progressing HIV disease stage and among PLWDH who were not virally suppressed at their last test. Antiretroviral (ARV) treatments thus may have protective effects which reduce the risk of severe COVID-19 among virally suppressed PLWDH. A recent study from Spain with a cohort of PLWDH receiving ARVs further suggests that tenofovir disoproxil fumarate, in particular, may lower risk for hospitalization and death [24].

Using a medical record review and retrospective matched cohort approach, we compared the demographics, clinical characteristics at hospital admission, comorbidities and clinical outcomes among PLWDH in NYS compared to HIV-negative controls who were hospitalized because of COVID-19. Among PLWDH, we also assessed the impact of HIV treatment regimen, viral load suppression, CD4 count, and other HIV-specific health indicators on clinical outcomes.

## Methods

### Study population and data sources

We conducted a retrospective matched cohort study of COVID-19 hospitalized individuals diagnosed between March 10 and June 6, 2020 in NYS. Individuals were considered COVID-19 hospitalizations if they had an admission date 30 days or less after a positive COVID-19 test result, a positive COVID-19 test result during the hospital encounter period, or a positive COVID-19 test result three days or less after discharge [3].

Data were utilized from the following: 1) the NYS HIV surveillance registry, which receives name-based reports for all HIV-related laboratory tests conducted for individuals who reside in or receive HIV-related care in NYS [25]; 2) the NYS Electronic Clinical Laboratory Reporting System, an electronic system for secure and timely transmission of reportable clinical laboratory information; 3) the State Health Information Network for NY (SHIN-NY), a public health information exchange network connecting NYS health care institutions; 4) electronic medical records. The NYS Department of Health (DOH) institutional review board approved this study as exempt research not requiring informed consent. Data were deidentified prior to analysis.

### Matching process

Individuals diagnosed with HIV as of June 2020 who were hospitalized due to COVID-19 were matched 2:1 with HIV-negative controls based on age, sex at birth, admitting facility, and admission date (S1 Table). Medical records were requested from hospitals by the NYS DOH contracting organization and abstracted by a trained nurse team. Following abstraction, medical records which were missing admission, discharge dates, and other core clinical variables were excluded from analysis (see S1 Fig for the sample selection). PLWDH without analyzable controls, based on this exclusion, were rematched to available controls using the original matching criteria. PLWDH without analyzable controls following the second match were excluded.

### Measures

#### Basic patient characteristics

Sociodemographic variables included sex at birth (in SHIN-NY data), age at time of admission (abstracted from medical records), and race/ethnicity (non-Hispanic White individuals, non-Hispanic Black individuals, Hispanic individuals, or other [Asian, Hawaiian/Pacific Islander, American Indian/Alaska native, multiracial, or another group not specified]). For PLWDH, race/ethnicity was determined from the HIV surveillance registry due to a higher degree of completion compared to medical record data. For controls, race/ethnicity was determined from medical records and supplemented by SHIN-NY data when unavailable from medical records. Additionally, tobacco smoking history (never, current, and former smokers) and body mass index (BMI; <25 kg/m^2^ [normal or underweight], 25–29 kg/m^2^ [overweight], and 30 kg/m^2^ or greater [obese]) were abstracted from medical records.

#### Comorbidities and clinical features

Medical record abstractions included information about preexisting general, renal, and immune comorbidities; vital signs and clinical lab values measured within 24 hours of hospital admission; acute symptoms present at admission; and COVID-19 treatments administered during hospitalization (specific antibiotics, COVID-19-specific treatments, non-steroidal anti-inflammatory drugs [NSAIDS], other angiotensin receptor blockers, and angiotensin-converting enzyme inhibitors).

We created a dichotomous variable to assess clinically significant elevation in one or more inflammatory marker, defined by meeting at least one of the following criteria: LDH>225 U/L, CRP>80 mg/L, ferritin>307 ng/mL for females and 336 ng/mL for males, D-Dimer>500 ng/mL, IL-6>1.8 pg/mL, procalcitonin>0.10 ng/mL, ESR>20mm/hr for females and 15 mm/hr for males, and fibrinogen>400 mg/dL.

#### HIV-related characteristics among PLWDH

The following HIV-related variables were created: Time since HIV diagnosis (1–2 years, 3–5 years, 6–10 years, 11–15 years, and >15 years), and CD4-defined HIV disease stage (stage 1: ≥500 cells/mm^3^, stage 2: 200–499 cells/mm^3^, and stage 3: <200 cells/mm^3^), and viral suppression of <200 copies/mL, based on the most recent laboratory reporting to NYS HIV surveillance within three years. HIV treatment regimen was abstracted from medical records and grouped into five mutually-exclusive categories (regimen containing both tenofovir and protease inhibitor; regimen with tenofovir, but no protease inhibitor; regimen with protease inhibitor, but not tenofovir; other ARV regimen; and no evidence of current ARV prescription prior to hospitalization), excluding regimens initiated within five days prior to or during hospitalization.

#### Outcomes

Medically abstracted clinical outcomes included the highest level of oxygen support needed (none, standard nasal cannula, high flow nasal cannula, and mechanical ventilation or extracorporeal membrane oxygenation [ECMO]), mechanical ventilation or ECMO, intensive care unit (ICU) admission, in-hospital death, and length of hospital stay.

### Analysis

Frequencies of patient demographics, comorbidities, clinical features at admission, and hospital outcomes were calculated for PLWDH and controls, and compared with Wald tests obtained from conditional (stratified) logistic regression models controlling for matched sets, with corresponding matched prevalence-ratios (mPR) (for conditions at admission) and risk-ratios (mRR) (for hospital outcomes) estimated by Mantel-Haenszel estimators, stratified on matched sets [26]. Among PLWDH, we examined the distribution of HIV-related characteristics and their associations with hospital outcomes, using χ^2^ tests. All results were deemed statistically significant at *P* <0.05.

## Results

### Study population

Medical records were requested for 935 PLWDH. Matched controls were selected from 1,750 possible (mean 1.9 controls per PLWDH) using the most stringent matching criteria (S1 Table). Of these 2,685 records requested, 2,627 were received and reviewed. Following removal of ineligible records and a second match, 853 PLWDH and 1,621 controls were available for analysis (S1 Fig).

The study sample was hospitalized in 86 facilities, with 33.2% (283) of PLWDH admitted in March, 55.8% (476) in April, 10.1% (86) in May, 0.9% (8) in June 2020. The majority of PLWDH (63.7%) and controls (62.9%) were diagnosed with COVID-19 upon hospital admission, an additional 16.3% of PLWDH and 16.4% of controls were diagnosed prior to hospital admission, 19.7% of PWLDH and 20.7% of controls were diagnosed during the course of their hospitalization, and 0.4% of PLWDH and 0.1% of controls were diagnosed the day following their discharge. Compared to controls, PLWDH more likely to be non-White, have a smoking history, and lower BMI (Table 1).

**Table 1:** Patient characteristics at admission among PLWDH and matched controls.

	PLWDH (n = 853)		Controls (n = 1,621)		Matched *P*-value
	*col %*	*n*	*col %*	*n*	
**Sex at birth**					--^a^
Female	29.2	249	28.9	469	
Male	70.8	604	71.1	1,152	
**Age** *(years)*					--^a^
22–29	1.4	12	0.9	15	
30–39	5.2	44	4.3	69	
40–49	11.0	94	11.4	184	
50–59	28.4	242	28.9	469	
60–69	33.7	287	33.7	546	
70–79	16.6	142	16.7	271	
80–91	3.8	32	4.1	67	
**Race/Ethnicity** ^b^					<0.0001
Non-Hispanic White	9.5	81	20.4	305	
Non-Hispanic Black	40.9	348	32.3	483	
Hispanic	41.4	353	36.5	545	
Other race/ethnicity	8.2	70	10.8	162	
*Unknown*		*1*		*126*	
**Smoking history**					< .0001
Never	55.8	415	70.0	964	
Former	29.8	222	23.0	317	
Current	14.4	107	7.0	96	
*Unknown*		*109*		*244*	
**BMI**					< .0001
< 25 kg/m^2^ (normal or underweight)	36.0	231	23.0	305	
25–29 kg/m^2^ (overweight)	31.3	201	33.2	440	
30+ kg/m^2^ (obese)	32.7	210	43.7	579	
*Unknown*		*211*		*297*	

Abbreviations: PLWDH, persons living with diagnosed HIV; BMI, body mass index.

^a^*P*-values not calculated due to matching on sex and age, by design.

^b^For PLWDH, race/ethnicity determined per the HIV surveillance registry due to higher degree of completion. For Controls, determined from the medical record, supplemented by the SHIN-NY record, if missing.

### Comorbidities

Histories of comorbidities significantly differed between PLWDH and controls (Table 2). Relative to controls, PLWDH were 1.22 (mPR [95% CI, 1.07–1.40]) times as likely to have cardiovascular disease, 6.71 (95% CI, 4.75–9.48) times as likely to have chronic liver disease, and 1.76 (95% CI, 1.40–2.21) times as likely to have chronic lung disease, but less likely to have hypertension (0.92 [95% CI, 0.86–0.98]) and diabetes mellitus (0.86 [95% CI, 0.78–0.96]). Similarly, PLWDH were 1.77 (95% CI, 1.50–2.09) times as likely to have a history of renal disease, including significantly higher levels of renal insufficiency, end stage renal disease, and a need for dialysis. No significant differences were observed in non-HIV immune conditions.

**Table 2:** Comorbidities noted at admission among PLWDH and matched controls.

	PLWDH		Controls		Matched	Matched Prevalence-Ratio	
	(n = 853)		(n = 1,621)		*P*-value		
	*col %*	*n*	*col %*	*n*		*mPR*	*(95% CI)*
**General conditions**							
**Any general conditions**	81.2	693	78.2	1,268	0.02	1.05	(1.01–1.09)
Hypertension	57.8	493	64.2	1,040	0.01	0.92	(0.86–0.98)
Diabetes Mellitus	36.8	314	42.9	696	0.004	0.86	(0.78–0.96)
Cardiovascular disease	29.0	247	23.8	386	0.004	1.22	(1.07–1.40)
Chronic liver disease, excluding cirrhosis	16.5	141	2.5	41	<0.0001	6.71	(4.75–9.48)
Asthma	15.8	135	12.2	197	0.01	1.30	(1.07–1.59)
Chronic lung disease, excluding asthma	13.8	118	8.0	129	<0.0001	1.76	(1.40–2.21)
Dementia	6.8	58	5.1	83	0.08	1.31	(0.97–1.76)
Cirrhosis	3.4	29	1.1	18	0.0001	2.97	(1.72–5.13)
Coagulopathy	2.2	19	2.6	42	0.52	0.84	(0.50–1.42)
Pregnancy	0.4	3	0.4	6	0.89	0.92	(0.36–2.40)
**Renal conditions**							
**Any renal** ^a^	25.2	215	14.0	227	<0.0001	1.77	(1.50–2.09)
Chronic renal insufficiency/ Chronic kidney disease	15.9	136	8.5	138	<0.0001	1.83	(1.47–2.27)
End stage renal disease	12.4	106	6.0	97	<0.0001	2.06	(1.59–2.68)
Dialysis	11.0	94	5.1	82	<0.0001	2.17	(1.63–2.90)
**Immune conditions**							
**Any immune** ^b^ ^,^ ^c^	10.6	90	8.4	136	0.07	1.26	(0.98–1.62)
Solid organ malignancy	4.5	38	3.9	63	0.48	1.15	(0.78–1.69)
Immunosuppressive therapy	2.6	22	2.2	35	0.37	1.27	(0.75–2.13)
Solid organ transplant	2.6	22	1.5	25	0.06	1.71	(0.97–3.00)
Metastatic cancer	1.3	11	1.2	20	0.90	0.95	(0.47–1.95)
Lymphoma	1.3	11	0.7	11	0.12	1.87	(0.85–4.11)
Steroid therapy	1.2	10	0.9	15	0.57	1.26	(0.56–2.80)
Immunoglobulin deficiency/ Immunodeficiency	0.5	4	0.2	3	0.20	2.67	(0.60–11.91)
Leukemia	0.4	3	0.5	8	0.58	0.69	(0.19–2.54)

Abbreviation: PLWDH, persons living with diagnosed HIV; mPR, matched prevalence-ratio.

^a^Additional renal conditions only found among controls included glomerulonephritis (n = 2), polycystic kidney disease (n = 4), and nephrotic syndrome (n = 1).

^b^Additional immune conditions only found among controls included multiple myeloma (n = 11), stem cell transplant (n = 5).

^c^Additional immune conditions sought in medical records but not found among PLWDH or controls included: complement deficiency and Grafts-Vs-Host disease.

### Clinical characteristics at admission

There were no significant differences between PLWDH and controls with respect to an elevated heart or respiratory rate, or low blood pressure (Table 3). PLWDH were significantly less likely to have fever (mPR, 0.80 [95% CI, 0.68–0.93]) and 0.91-fold (95% CI, 0.77–1.07) O_2_ saturation <90% relative to >93% (mPR, 0.91 [95% CI, 0.77–1.07]). PLWDH were significantly more likely to report experiences of diarrhea (mPR, 1.28 [95% CI, 1.07–1.54]), altered mental status (mPR, 1.46 [95% CI, 1.20–1.79]), and upper-respiratory infection (mPR, 5.54 [95% CI, 1.88–16.34]).

**Table 3:** Vital signs, lab values, and symptoms at admission among PLWDH and matched controls^a^.

	PLWDH		Controls		Matched *P*-value	Matched Prevalence-Ratio	
	(n = 853)		(n = 1,621)				
	*col %*	*n*	*col %*	*n*		*mPR*	*(95% CI)*
**Vital signs**							
Elevated heart rate^b^	24.1	205/851	25.3	409/1,618	0.57	0.96	(0.83–1.11)
Elevated respiratory rate^c^	26.0	221/851	26.3	425/1,616	0.96	1.00	(0.88–1.15)
Low blood pressure^d^	10.3	88/852	10.2	165/1,621	0.91	1.01	(0.80–1.29)
Fever^e^	19.5	166/850	24.7	400/1,619	0.004	0.80	(0.68–0.93)
O_2_ saturation					0.046		
<90%	19.9	169/850	23.8	385/1,616		0.91	(0.77–1.07)
90–93%	21.4	182/850	18.4	297/1,616		1.14	(0.96–1.35)
>93%	58.7	499/850	57.8	934/1,616		ref	
**Clinical lab values**							
Elevated hematocrit^f^	4.5	38/843	4.8	78/1,613	0.95	0.99	(0.68–1.44)
Elevated platelet count^g^	4.0	34/840	3.6	58/1,614	0.58	1.12	(0.74–1.71)
Low platelet count^h^	25.4	213/840	20.0	323/1,614	0.001	1.30	(1.12–1.51)
Elevated sodium^i^	6.9	58/844	6.1	99/1,610	0.39	1.14	(0.85–1.52)
Elevated BUN^j^	48.6	409/842	41.6	670/1,610	0.0004	1.17	(1.08–1.28)
Elevated creatinine^k^	43.3	365/843	33.5	540/1,610	<0.0001	1.31	(1.18–1.44)
Elevated glucose^l^	73.0	616/844	84.3	1,357/1,609	<0.0001	0.87	(0.83–0.91)
Elevated AST^m^	55.4	445/803	59.6	918/1,541	0.08	0.94	(0.87–1.01)
Elevated ALT^n^	31.3	252/804	40.0	621/1,553	<0.0001	0.79	(0.70–0.89)
**Inflammatory markers**							
Elevated white blood cell count^o^	23.3	196/841	30.1	486/1,614	0.0003	0.77	(0.67–0.89)
Elevated LDH^p^	86.9	564/649	90.9	1,156/1,272	0.01	0.96	(0.92–0.99)
Elevated CRP^q^	37.4	247/660	43.2	573/1,326	0.03	0.89	(0.81–0.99)
Elevated ferritin^r^	73.4	481/655	78.9	1,017/1,289	0.004	0.93	(0.88–0.98)
Elevated D-dimer^s^	69.7	419/601	72.9	881/1,208	0.26	0.97	(0.91–1.02)
Composite: At least one elevated marker^t^	95.2	746/784	95.1	1,423/1,499	0.47	1.01	(0.99–1.03)
**Symptoms recorded** ^u^							
Shortness of breath/respiratory distress	65.7	560	64.4	1,044	0.34	1.03	(0.97–1.09)
Cough	56.7	484	60.3	978	0.19	0.96	(0.9–1.02)
Fever/chills	54.4	464	60.5	980	0.01	0.91	(0.85–0.98)
Hypoxia	22.2	189	22.7	368	0.71	0.97	(0.84–1.13)
Myalgia	20.0	171	18.3	296	0.19	1.12	(0.95–1.31)
Diarrhea	18.9	161	14.9	241	0.01	1.28	(1.07–1.54)
Altered mental status/confusion	15.6	133	10.3	167	0.0003	1.46	(1.2–1.79)
Nausea/vomiting	14.1	120	12.0	194	0.20	1.15	(0.93–1.41)
Chest pain	11.0	94	10.5	170	0.74	1.04	(0.82–1.33)
Abdominal pain	8.6	73	6.4	104	0.12	1.26	(0.94–1.67)
Headache	6.6	56	7.3	118	0.45	0.89	(0.66–1.2)
Congestion	3.8	32	3.0	48	0.23	1.31	(0.84–2.04)
Sore throat	3.4	29	3.5	57	0.86	0.96	(0.62–1.48)
Loss of taste	2.5	21	1.7	28	0.29	1.35	(0.77–2.37)
Loss of smell	1.8	15	0.8	13	0.06	1.97	(0.97–3.98)
Upper-respiratory infection/influenza-like illness	1.5	13	0.3	5	0.002	5.54	(1.88–16.34)
Rash	0.5	4	0.2	3	0.25	2.29	(0.56–9.25)
Wheezing	0.4	3	1.2	19	0.06	0.31	(0.09–1.03)
Seizures	0.4	3	0.4	7	0.73	0.80	(0.21–2.98)
Blood clots^v^	0.4	3	0.4	7	0.70	0.77	(0.21–2.82)
Hemoptysis	0.4	3	0.4	7	0.74	0.80	(0.21–2.98)

Abbreviations: PLWDH, persons living with diagnosed HIV; mPR, matched prevalence-ratio; BUN, blood urea nitrogen; AST, aspartate transaminase; ALT, alanine aminotransferase; LDH, lactate dehydrogenase; CRP, c-reactive protein.

^a^Excludes vital signs/ laboratory values with less than 70% completion among PLWDH: IL-6 (82%), Glasgow Coma Scale (76%), fibrinogen (75%), arterial pH (72%), ESR (71%), and procalcitonin (41%).

^b^Elevated heart rate: > 110 beats per minute.

^c^Elevated respiratory rate: > 22 breaths per minute.

^d^Low blood pressure rate: systolic blood pressure < 90 mmHG or diastolic blood pressure < 60 mmHG.

^e^Fever: temperature > 38.0 degrees Celsius.

^f^Elevated hematocrit level: > 47% for females, > 50% for males.

^g^Elevated platelet count: > 450 cells x 10^9^/L.

^h^Low platelet count: < 150 cells x 10^9^/L.

^i^Elevated sodium level: > 145 mmol.

^j^Elevated BUN level: > 20 mg/dL.

^k^Elevated creatinine level: > 1.2 mg/dL for females, > 1.4 mg/dL for males.

^l^Elevated glucose level: > 99 mg/dL.

^m^Elevated AST level: > 40 U/L.

^n^Elevated ALT level: > 40 U/L.

^o^Elevated white blood cell count: > 9.6 cells x 10^9^/L.

^p^Elevated LDH level: > 225 U/L.

^q^Elevated CRP level: > 0.8 mg/dL.

^r^Elevated ferritin level: > 307 ng/mL for females, 336 ng/mL for males.

^s^Elevated D-dimer level: > 500 ng/mL.

^t^Elevated inflammatory marker was defined by meeting at least one of the following criteria: LDH > 225 U/L, CRP > 80 mg/L, ferritin > 307 ng/mL for females and 336 ng/mL for males, D-Dimer > 500 ng/mL, IL-6 > 1.8 pg/mL, procalcitonin > 0.10 ng/mL, ESR > 20mm/hr for females and 15 mm/hr for males, and fibrinogen > 400 mg/dL.

^u^Additional symptom reported only among PLWDH included conjunctivitis (n = 1).

^v^Blood clots include deep vein thrombosis, pulmonary embolism, and thrombosis.

Initial clinical laboratory values differed significantly between PLWDH and controls, as PLWDH were more likely to have low platelets (mPR, 1.30 [95% CI, 1.12–1.51]), elevated BUN (mPR, 1.17 [95% CI, 1.08–1.28]), and creatinine (mPR, 1.31 [95% CI, 1.18–1.44]), but less likely to have elevated glucose (mPR, 0.87 [95% CI, 0.83–0.91]), AST (mPR, 0.94 [95% CI, 0.87–1.01]), and ALT (mPR, 0.79 [95% CI, 0.70–0.89]), consistent with observed histories of renal disease and diabetes mellitus (Table 3). When assessed, PLWDH were less likely to have elevated white blood cell count (mPR, 0.77 [95% CI, 0.67–0.89]), LDH (mPR, 0.96 [95% CI, 0.92–0.99]), CRP (mPR, 0.89 [95% CI, 0.81–0.99]), and ferritin (mPR, 0.93 [95% CI, 0.88–0.98]) but were equally likely to have at least one elevated inflammatory marker.

### Therapies

S2 Table summarizes medications administered to patients during hospitalization. The most common medications prescribed were azithromycin (55.5% PLWDH, 60.5% controls), vancomycin (30.5% PLWDH, 28.4% controls), ceftriaxone (46.0% PLWDH, 50.0% controls), hydroxychloroquine (60.5% PLWDH, 66.1% controls), and acetaminophen (60.8% PLWDH, 78.6% controls).

### Outcomes

Major outcomes of hospitalization are presented in Table 4. Relative to controls, PLWDH were significantly less likely to require mechanical ventilation or ECMO (21.6% vs. 25.7%; mRR, 0.85 [95% CI, 0.74–0.99]) and admission to ICU (23.6% vs. 27.8%; mRR, 0.85 [95% CI, 0.74–0.98]). No significant differences were found in in-hospital deaths between PLWDH and controls (23.4% vs. 25.5%; mRR, 0.94 [95% CI, 0.82–1.08]). No significant differences were found in the length of hospital stay between PLWDH and controls overall (median 6 vs. 7 days, respectively, *P* = 0.39), among those patients who were alive at discharge, or among those patients who died.

**Table 4:** Hospitalization outcomes among PLWDH and matched controls.

	PLWDH		Controls		Matched *P*-value	Matched Risk-Ratio	
	(n = 853)		(n = 1,621)				
	*col %*	*n*	*col %*	*n*		*mRR*	*(95% CI)*
**Highest level of O**_**2**_ **support administered**					0.04	--	
No support	22.2	189/852	17.9	290/1,620		REF	
Nasal cannula	33.5	285/852	35.7	587/1,620		0.89	(0.81–0.99)
HFNC	19.0	162/852	16.8	272/1,620		0.91	(0.76–1.09)
BiPAP or CPAP	3.9	33/852	4.1	67/1,620		1.12	(0.69–1.81)
Mechanical ventilation/ECMO	21.5	183/852	25.5	413/1,620		0.97	(0.84–1.11)
**Mechanical ventilation/ECMO**	21.6	183/847	25.7	413/1,610	0.04	0.85	(0.74–0.99)
**ICU**	23.6	201	27.8	451	0.02	0.85	(0.74–0.98)
**Death**	23.4	200	25.5	414	0.37	0.94	(0.82–1.08)
	*Median (Q1*, *Q3) [Min*, *Max]*		*Median (Q1*, *Q3) [Min*, *Max]*		*Wilcoxon P-value*		
**Length of Stay**							
Overall	6 (3, 12) [0, 117]		7 (3, 12) [0, 145]		0.39		
Among discharged alive	6 (3, 11) [0, 117]		6 (3, 11) [0, 145]		0.63		
Among died	8 (4, 14) [0, 66]		8 (4, 15) [0, 130]		0.55		

Abbreviations: PLWDH, persons living with diagnosed HIV; mRR, matched risk-ratio; HFNC, high flow nasal cannula; BiPAP, bilevel positive airway pressure; CPAP, continuous positive airway pressure; ECMO, extracorporeal membrane oxygenation; ICU, intensive care unit.

### HIV-related characteristics

The majority of PLWDH (88.2%) had been diagnosed with HIV for more than 10 years, were virally suppressed (87.9%), and were classified as HIV disease Stage 1 (51.2%) (Table 5). No significant differences were observed in the likelihood of mechanical ventilation/ECMO, ICU admission, or in-hospital death by time since HIV diagnosis, HIV disease stage, or viral suppression.

**Table 5:** Distribution of and hospital outcomes associated with HIV-related characteristics among PLWDH hospitalized with COVID-19.

	Distribution		Mechanical Ventilation/ECMO			ICU			Death		
	*col %*	*n*	*row %*	*n*	*P-value*	*row %*	*n*	*P-value*	*row %*	*n*	*P-value*
**Overall**			21.6	183/847		23.6	201		23.4	200	
**Time since HIV Diagnosis**					0.47			0.69			0.09
1–2 years	2.1	18	5.6	1/18		11.1	2		16.7	3	
3–5 years	2.7	23	17.4	4/23		17.4	4		17.4	4	
6–10 years	6.9	59	18.6	11/59		22.0	13		15.3	9	
11–15 years	11.1	94	22.3	21/94		24.5	23		16.0	15	
>15 years	77.1	655	22.3	145/649		24.1	158		25.8	169	
**HIV Disease Stage at last CD4 Test**, *in prior 3 years*					0.46			0.26			0.93
Stage 1 (≥500 cells/mm^3^)	51.2	418	23.2	96/413		24.9	104		22.7	95	
Stage 2 (200–499 cells/mm^3^)	34.8	284	20.5	58/283		23.2	66		23.9	68	
Stage 3 (<200 cells/mm^3^)	14.0	114	18.4	21/114		17.5	20		23.7	27	
**Viral Suppression at last test**, *in prior 3 years*					0.90			0.43			0.57
Suppressed (<200 copies/mL at last test)	87.9	718	21.8	155/712		23.8	171		23.8	171	
Not suppressed	12.1	99	21.2	21/99		20.2	20		21.2	21	
**Current HIV ARV prescription regimen** ^a^					0.14			0.52			0.22
Tenofovir-containing regimens	66.3	496	19.1	94/492	0.02^b^	22.6	112	0.27^b^	20.6	102	0.03^b^
Regimen with tenofovir and protease inhibitor	10.2	87	16.1	14/87		21.8	19		19.5	17	
Regimen with tenofovir, but no protease inhibitor	47.9	409	19.8	80/405		22.7	93		20.8	85	
Non- tenofovir -containing regimens	33.7	252	26.3	66/251		26.2	66		27.8	70	
Regimen with protease inhibitor, but not tenofovir	11.8	101	22.8	23/101		21.8	22		28.7	29	
Other ARV regimen	17.7	151	28.7	43/150		29.1	44		27.2	41	
No evidence of current ARV prescription	12.3	105	22.1	23/104		21.9	23		26.7	28	

Abbreviations: PLWDH, persons living with diagnosed HIV; ECMO, extracorporeal membrane oxygenation; ICU, intensive care unit; ARV, antiretroviral medication.

^a^Current regimen defined as evidence of ARV regimen 5 or more days prior to hospitalization. Tenofovir-containing ARVs included Atripla, Biktarvy, Cimduo, Complera, Delstrigo, Descovy, Genvoya, Odefsey, Stribild, Symfi, Symfi Lo, Symtuza, Truvada, and Viread. Protease inhibitor-containing ARVs included Aptivus, Crixivan, Evotaz, Invirase, Kaletra, Lexiva, Norvir, Prezcobix, Prezista, Reyataz, Symtuza, Tybost, and Viracept.

^b^Relative to non-tenofovir-containing regimens.

Eighty-eight percent of PLWDH had evidence of a current ARV prescription (Table 5). Sixty-six percent had evidence of a tenofovir-containing regimen. PLWDH prescribed tenofovir-containing regimen were significantly less likely to receive mechanical ventilation/ECMO (19.1% vs. 26.3%; RR, 0.73 [95% CI, 0.55–0.96]) or die in hospital (20.6% vs. 27.8%; RR, 0.74 [95% CI, 0.57–0.96]), relative to PLWDH prescribed non-tenofovir-containing regimens.

## Discussion

This study retrospectively reviewed medical records to comprehensively describe whether and how COVID-19 clinical profiles and outcomes differ between PLWDH and a matched cohort of non-PLWDH early in the COVID-19 pandemic. We found no significant difference in in-hospital mortality and lower likelihoods of receiving mechanical ventilation/ECMO or ICU admission in PLWDH relative to controls. Interestingly, self-reported symptoms or vital signs did not show evidence that PLWDH presented with worse COVID-19 illnesses upon hospitalization. PLWDH had lower prevalence of fever and elevated inflammatory markers and trended towards better vital signs at admission compared to controls. However, consistent with prior reports [11, 12, 16, 23], PLWDH in our study were significantly more likely to have chronic conditions associated with severe COVID-19 illnesses, including chronic lung disease, chronic liver disease, cardiovascular disease, and renal conditions. Despite higher prevalence of these comorbidities, PLWDH were not more likely to show poorer COVID-19 outcomes. Examination of medications received during hospitalization did not indicate that PLWDH received more intensive COVID-19 treatment relative to controls.

While results align with another study [27] which found lower rates of mechanical ventilation and mortality among hospitalized PLWDH in New York City early in the pandemic, they contrast with other reports of significantly higher in-hospital mortality among PLWDH relative to non-PLWDH [18, 28]. This contrast may be partly explained due to our use of matching on key characteristics of age, sex, and admission facility and date. These matching variables were selected because they have been associated with hospitalization and more severe illness due to COVID-19 [3, 16, 18] and allowed for a critical exploration of the impact of comorbidities on COVID-19.

The findings from the present study may also explain higher hospitalization rates reported in two studies that utilized surveillance data [3, 16]. PLWDH with COVID-19 may not be hospitalized due to presentation of more severe COVID-19 symptoms; rather, HIV-related immunocompromise associated with co-existing comorbidities may result in decision for more careful monitoring at the hospital. The presence of comorbidities is considered a potential pathway to severe COVID-19 illnesses in PLWDH [13, 17]. Although speculative, our findings support that intervention at the hospital before symptoms worsen may reduce risk for development of severe COVID-19 outcomes. Lower prevalence of mechanical ventilation and ICU admission in PLWDH relative to controls may also be explained due to the relatively high levels of viral suppression in the study population, particularly since other studies have found worse outcomes among PLWDH who are not on HIV treatment, virologically unsuppressed, or with CD4 below 200 [19, 22, 23].

While risks for hospitalization among PLWDH increase with more advanced HIV disease stage [3], we did not find any significant differences in hospitalization outcomes among PLWDH by viral suppression status or HIV disease stage. This is consistent with our earlier findings that once hospitalized, there are no differences in mortality between PLWDH and non-PLWDH [3].

Among patients with evidence of ARV prior to hospitalization, those on tenofovir-containing regimens experienced less mechanical ventilation and death compared to those on non-tenofovir-containing regiments. This is consistent with other studies demonstrating the potentially protective value of tenofovir [24, 29, 30]. Additionally, there is evidence that the use of tenofovir-containing regimens was underrepresented among our hospitalized sample of PLWDH, suggesting the possibility that those on tenofovir may have been less likely to be hospitalized due to COVID. The 66.3% of patients on tenofovir-containing regimen in our sample was lower than two other recent community samples (69.6% [7,054/10,128; *P* = 0.06] of NYS DOH AIDS Drug Assistance Program [31] ARV claims during January-March 2020, and 80.1% [2,116/2,642, *P*<0.0001] of records reviewed for the NYS Medicaid HIV Special Needs Plans 2019 quality of care review, which included 2,669 enrollees from three NYS Managed Care Plans and was queried for the 2,642 patients who were prescribed ARVs at any time during 2019; comparisons were evaluated using χ^2^ tests). More research is needed to systematically investigate this possibility.

### Limitations

This study focuses on COVID-19 cases identified early in the pandemic in NYS, driven by the original variant, where poor outcomes were likely more frequent due to lack of knowledge about the disease, minimal use of effective therapies, and unavailability of vaccines. Therefore, these findings may be less generalizable to PLWDH in the current era of COVID-19 or outside NYS, however, these findings provide a clear clinical picture of the natural history of COVID-19 among PLWDH and focused on the epicenter of COVID-19 at the time. Medical record abstractions did not include a history of treatment for comorbidities, which prevented accounting for severity or progression of these conditions. While our study did not find significantly elevated risk for severe COVID-19 outcomes among hospitalized PLWDH, this study is unable to definitively determine whether there was a lower threshold to hospitalization for PLWDH. Analyses were limited to availability of results from public health reporting of HIV-related laboratory tests reported to HIV surveillance; many people did not undergo viral load and CD4 testing upon admission, which limited the understanding of severe COVID-19 on these measures. We also were limited to information on ARV regimen noted in medical records, which may not reflect adherence to the medication or non-reporting of ARV prescription at the time of hospitalization. The relationship between tenofovir-containing regimens and better hospitalization outcomes may be spurious and due to relationships not controlled for in our analysis. Additionally, we utilized two recent subpopulations of PLWDH to identify community ARV rates: PLWDH served by the NYS DOH AIDS Drug Assistance Program and PLWDH served by Medicaid HIV Special Needs Plans. Both subpopulations represent persons of lower socioeconomic status. Thus, they may not be fully reflective of all PLWDH within NYS.

## Conclusion

The present study provides evidence of the importance of comorbidities on severe COVID-19 outcomes for PLWDH. Additional findings include the potential protective effect of tenofovir and lower rates of elevated inflammatory markers in PLWDH. While mortality did not differ among hospitalized PLWDH and controls in this study, our previous study [3] found that PLWDH were more likely to be hospitalized with COVID-19. Per CDC recommendations [2], immunocompromised persons are recommended to stay up-to-date with vaccinations. Given these earlier findings, it remains essential to prioritize persons with any immunocompromised condition, including PLWDH, for full vaccination, with additional and booster doses as eligible, to prevent severe COVID-19 disease and hospitalization.

## Supporting information

S1 Table Summary of matching process. (PDF)

S2 Table Distribution of COVID-19 treatments among PLWDH and matched controls. (PDF)

S1 Fig Flowchart: Records available for analysis. (PDF)
