# Supplementary material for: Factors associated with SARS-CoV-2-related hospital outcomes among and between persons living with and without diagnosed HIV infection in New York State
Source: PLoS One. 2022 May 25;17(5):e0268978. doi: 10.1371/journal.pone.0268978 (PMC9132290; doi:10.1371/journal.pone.0268978)
Supplement: S1 Table — (PDF) [file pone.0268978.s001.pdf]

S1 Table. Summary of matching process

Criteria for controls, relative to each PLWDH					Number selected	
**Level**	**Sex at birth**	**Age**	**Admission**	**Facility name**	**PLWDH**	**Controls**
1	Exact	±5 years	±3 days	Exact	849	1,621
2	Exact	±5 years	±7 days	Exact	49	72
3	Exact	±5 years	±14 days	Exact	23	36
4	Exact	±10 years	±14 days	Exact	14	21
**Total**					935	1,750

Abbreviation: PLWDH, persons living with diagnosed HIV.
