# Supplementary material for: Factors associated with SARS-CoV-2-related hospital outcomes among and between persons living with and without diagnosed HIV infection in New York State
Source: PLoS One. 2022 May 25;17(5):e0268978. doi: 10.1371/journal.pone.0268978 (PMC9132290; doi:10.1371/journal.pone.0268978)
Supplement: S2 Table — (PDF) [file pone.0268978.s002.pdf]

**S2 Table. Distribution of COVID-19 treatments among PLWDH and matched controls**[a, b, c]

| | PLWDH (n = 853) | | Controls (n = 1,621) | | *Matched P* |
|---|---|---|---|---|---|
| | *col %* | *n* | *col %* | *n* | |
| **Antibiotics** | | | | | |
| Erythromycin: Erythrocin, Eryped, Ery-tab, E-Mycin | 0.1 | 1/847 | 0.4 | 7/1,605 | 0.18 |
| Clarithromycin: Biaxin, Biaxin XL | 0.2 | 2/846 | 0.1 | 1/1,605 | 0.21 |
| Azithromycin (Zithromax, Azithromycin, Z-Pack, Zmax) | 55.5 | 471/848 | 60.5 | 979/1,617 | 0.02 |
| Vancomycin | 30.5 | 258/847 | 28.4 | 458/1,613 | 0.26 |
| Piperracillin | 15.0 | 127/847 | 4.2 | 67/1,609 | <0.0001 |
| Ceftriaxone | 46.0 | 390/848 | 50.0 | 808/1,616 | 0.050 |
| Levofloxacin | 5.3 | 45/847 | 5.2 | 83/1,606 | 0.82 |
| **Specific COVID-19 therapies** | | | | | |
| Remdesivir | 2.8 | 24/847 | 2.3 | 37/1,605 | 0.50 |
| Hydroxychloroquine | 60.5 | 512/846 | 66.1 | 1,069/1,618 | 0.02 |
| Chloroquine | 0.8 | 7/847 | 0.1 | 2/1,606 | 0.02 |
| Tocilizumab | 3.4 | 29/847 | 7.3 | 117/1,611 | <0.0001 |
| Acetaminophen | 60.8 | 515/847 | 78.6 | 1,274/1,620 | <0.0001 |
| Convalescent Plasma Therapy | 3.5 | 30/847 | 3.2 | 52/1,606 | 0.87 |
| Dexamethasone | 3.4 | 29/847 | 5.7 | 91/1,609 | 0.01 |
| Other Steroids | 19.0 | 161/846 | 28.3 | 456/1,611 | <0.0001 |
| Other COVID-Directed Antiretroviral Therapies | 0.4 | 3/846 | 2.3 | 37/1,601 | 0.001 |
| **NSAIDS** | | | | | |
| Aspirin | 19.9 | 169/848 | 21.7 | 350/1,610 | 0.32 |
| Ibuprofen (Motrin, Advil) | 1.3 | 11/847 | 2.1 | 33/1,607 | 0.19 |
| **Other Angeotensin Receptor Blockers** | | | | | |
| Cozaar (Losartan) | 3.2 | 27/846 | 4.7 | 76/1,608 | 0.09 |
| Diovan (valsartan) | 0.4 | 3/846 | 0.6 | 10/1,608 | 0.34 |
| Prexartan (valsartan) | 0.2 | 2/846 | 0.1 | 2/1,607 | 0.49 |
| **ACE Inhibitors** | | | | | |
| Enalapril/Enalaprilat (vasotec oral and injectable) | 1.9 | 16/846 | 1.1 | 18/1,607 | 0.17 |
| Lisinopril (Zestril and Prinivil) | 4.5 | 38/846 | 4.9 | 78/1,605 | 0.80 |
| Ramipril (Altace) | 0.1 | 1/846 | 0.3 | 5/1,607 | 0.36 |

Abbreviations: PLWDH, persons living with diagnosed HIV; NSAIDS, non-steroidal anti-inflammatory drugs; ACE, angiotensin-converting enzyme.

[a] Additional treatments sought in medical records but not found among PLWDH or controls include: Sofosbuvir, Daclatasvir, Diclofenac (Cambia, Cataflam, Voltaren-XR, Zipsor, Zorvolex), Oxaprozin (Daypro), Avapro (irbesartan), Benicar (Olmesartan), Micardis (telmisartan), Teveten (eprosartan), Edarbi (azilsartan medoxomil), Benazepril (Lotensin), Moexipril (Univasc), Fosinoapril (Monopril), Perindopril (Aceon), Quinapril (Accupril), Trandolapril (Mavik).

[b] Additional treatments sought in medical records but found only among controls include: Celecoxib (Celebrex) (n=2), Indomethacin (Indocin) (n=1), Naproxen (Aleve, Anaprox, Naprelan, Naprosyn) (n=1), ATACAND (candesartan) (n=1).

[c] Additional treatments sought in medical records but found only among PLWDH include: Piroxicam (Feldene) (n=1), Captopril (Capotne) (n=1).
