# Supplementary material for: Factors associated with SARS-CoV-2-related hospital outcomes among and between persons living with and without diagnosed HIV infection in New York State
Source: PLoS One. 2022 May 25;17(5):e0268978. doi: 10.1371/journal.pone.0268978 (PMC9132290; doi:10.1371/journal.pone.0268978)
Supplement: S1 Fig — (PDF) [file pone.0268978.s003.pdf]

S1 Fig. Flowchart: records available for analysis

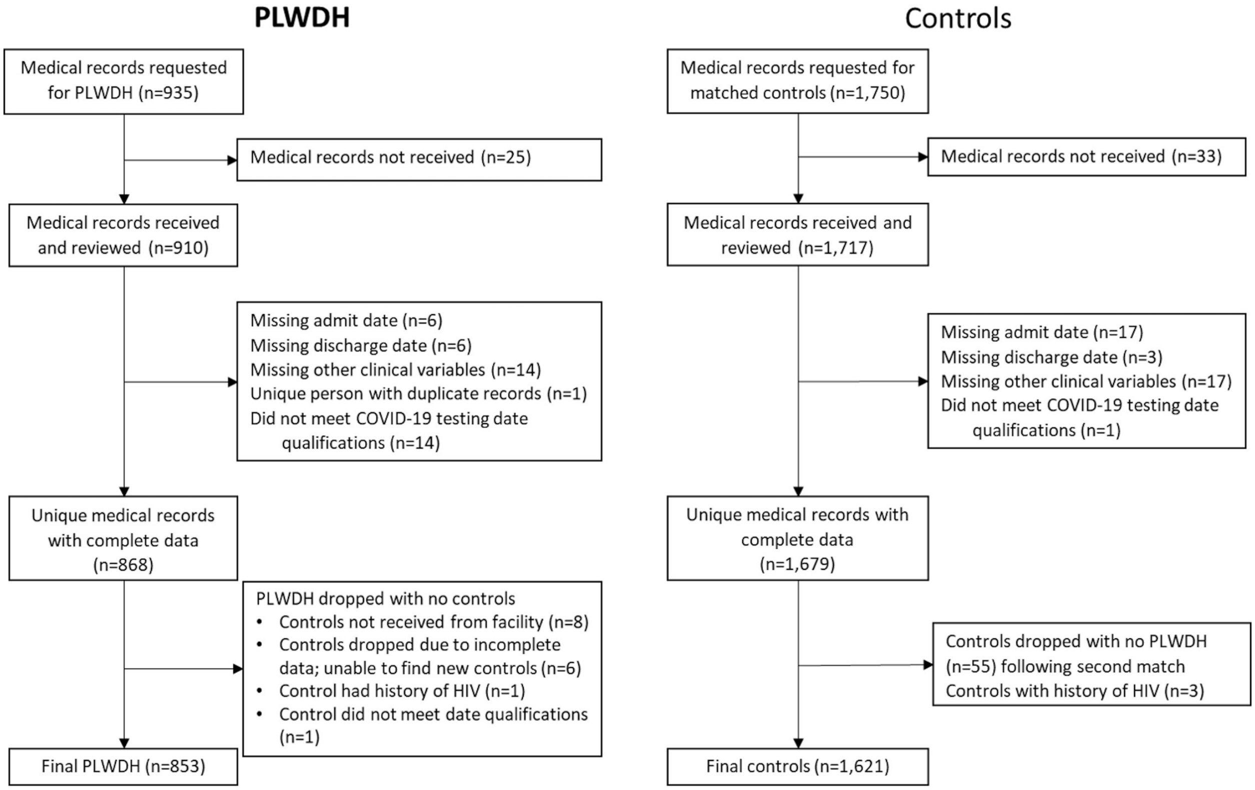

Abbreviations: PLWDH, persons living with diagnosed HIV.
